# Totally intramyocardial bullet in a clinically stable patient: a successfully managed case from a low income country

**DOI:** 10.1093/jscr/rjad013

**Published:** 2023-04-13

**Authors:** Desalegn Fekadu, Abebe Bezabih, Mahlet Tesfaye, Abdella Hayato, Natnael Muluneh, Segni Kejela

**Affiliations:** Department of Surgery, College of Health Sciences, Addis Ababa University, Addis Ababa, Ethiopia; Department of Surgery, College of Health Sciences, Addis Ababa University, Addis Ababa, Ethiopia; Department of Surgery, College of Health Sciences, Addis Ababa University, Addis Ababa, Ethiopia; Department of Surgery, College of Health Sciences, Addis Ababa University, Addis Ababa, Ethiopia; Department of Surgery, College of Health Sciences, Addis Ababa University, Addis Ababa, Ethiopia; Department of Surgery, College of Health Sciences, Addis Ababa University, Addis Ababa, Ethiopia

## Abstract

Intra-cardiac retained bullets rare entities in clinical practice owing to the high mortality associated with it. We present a case of a 26-year-old male patient presented 24 days after sustaining a bullet injury to the left side of the chest. Intra-operatively totally myocardial bullet was found within the right ventricle and extracted successfully.

## INTRODUCTION

Intracardiac retained bullets are rarely been reported owing the high-associated mortality rates among patients that sustained the injury and the immediate surgical intervention warranted in the vast majority that presented with such injuries [1, 2]. Here, we report a case of a 26-year-old male patient who presented over 3 weeks after sustaining bullet injury to the chest/heart.

## CASE REPORT

We report a case of a 26-year-old male patient who presented with bullet injury to the anterior chest 24 days prior to his presentation. The incident occurred while the patient was at home sitting and bullet penetrated through the roof of the house and injured his anterior chest. He immediately had bleeding from the wound, which was minimal and spontaneously stopped. He also had mild retrosternal chest pain but no shortness of breath or cough. The patient was subsequently taken to a local hospital where investigations were done and he was referred to our center for cardiovascular surgery consultation.

On physical examination, the patient appeared well, with vital signs, pulse rate of 68 beats/minute with a full volume and a regular rhythm. The respiratory rate was 16 breaths/minute. The blood pressure and oxygen saturation was also within the normal range. The pertinent physical finding was on the chest exam, which showed left parasternal fifth intercostal space healed 1 × 1 cm hypo-pigmented scar. Air entry was good, chest was clear bilaterally, and the cardiovascular examination was non-revealing.

**Figure 1:**
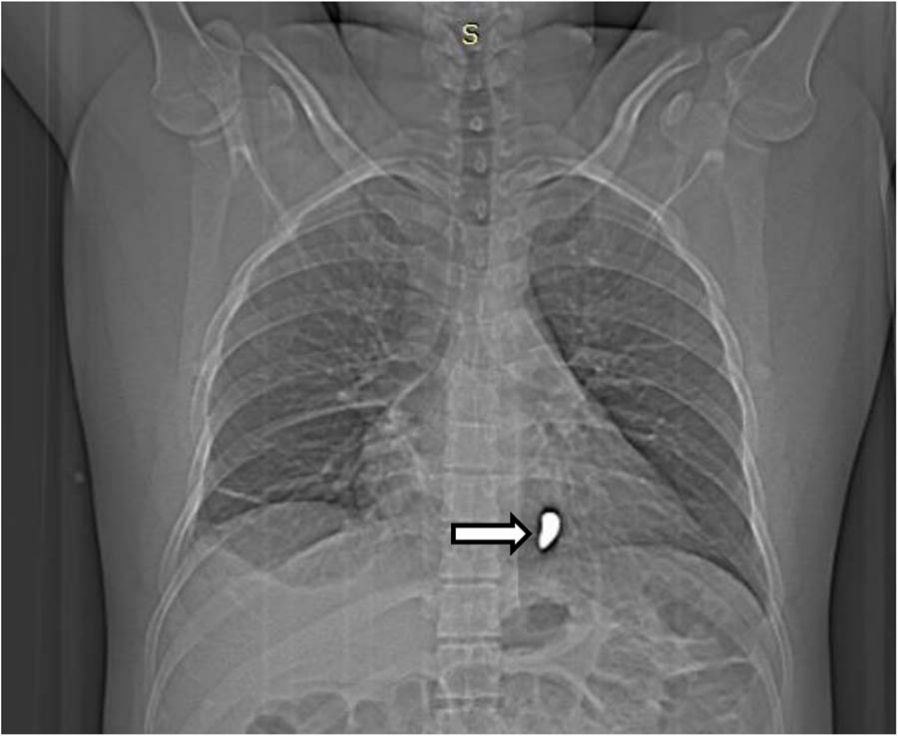
Scout image of the chest showing a radio-opaque foreign body in the left side of the chest (white arrow).

**Figure 2:**
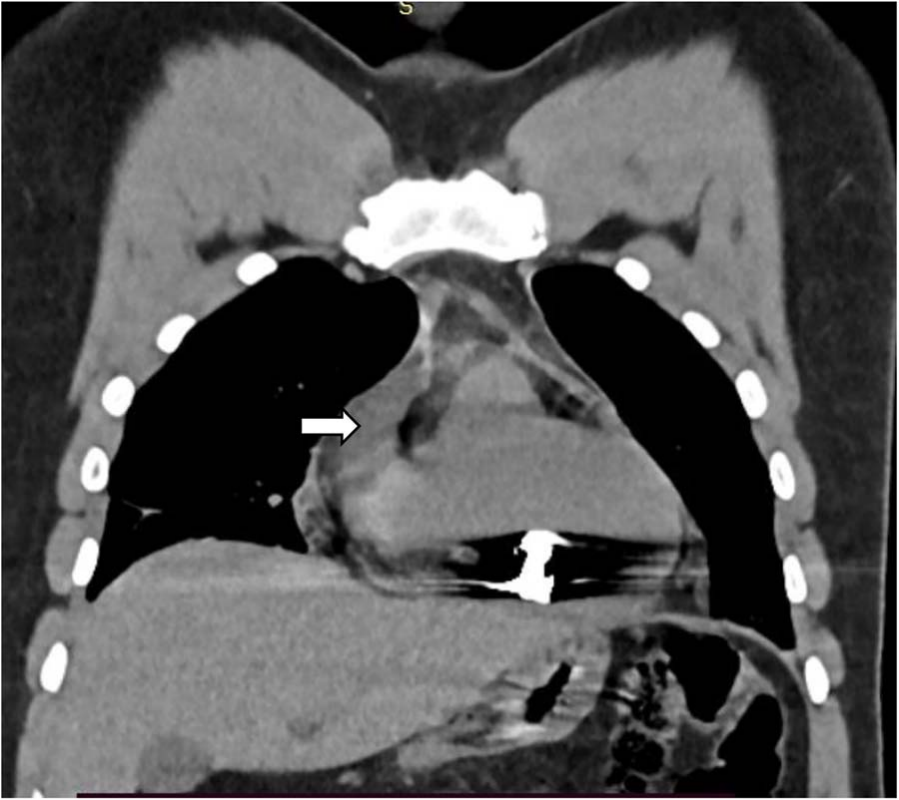
Coronal CT image showing foreign body with in the myocardium of the right ventricle (white arrow).

**Figure 3:**
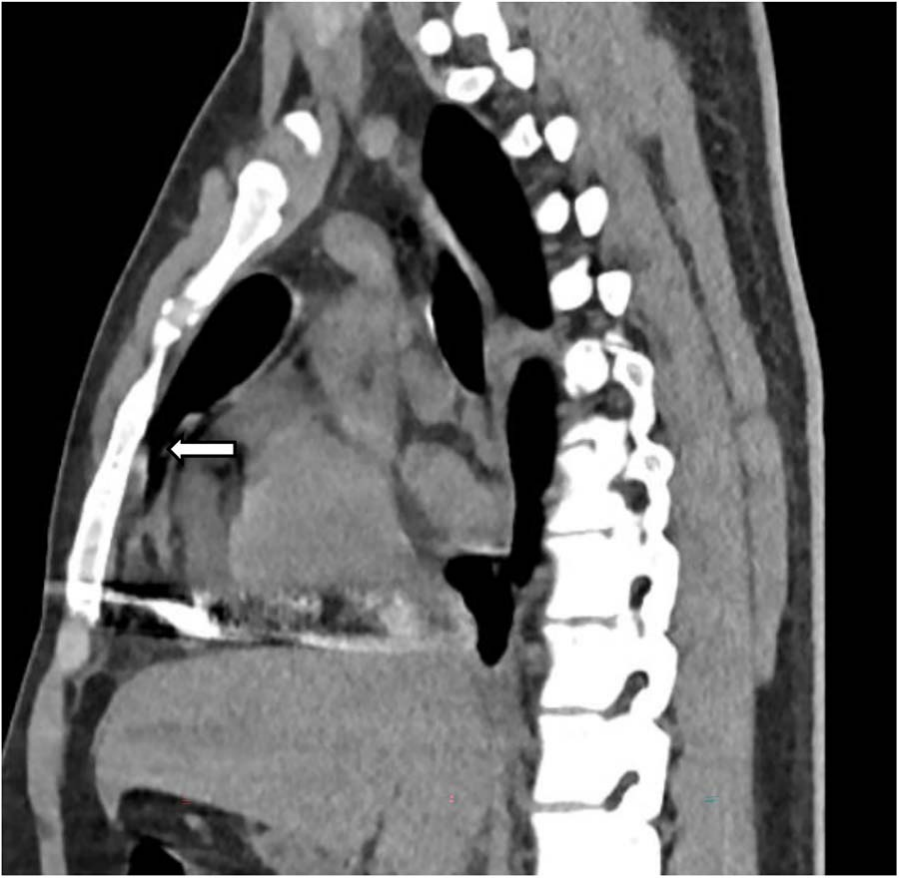
Sagittal image depicting foreign body within the myocardium (white arrow).

The complete blood count showed white cell count of 4300 cells, hemoglobin of 18.7 g/dl, and platelet of 285 000. Serum electrolyte, albumin and liver function tests were within normal range and creatinine was 1.19 mg/dl. Chest X ray showed bullet left of the sternum within the inferior aspect of the cardiac silhouette. Contrast CT scan detected minimal bilateral pleural collection with right basal segment atelectasis with 2.3 × 1.1 cm metallic density foreign body in the mediastinum located over the inferior border of ventricular wall (Figs 1–3). Troponin was 432.4 ng/ml. Electrocardiography showed V2 lead ST segment elevation. All the other leads were normal. The patient had three echocardiographs done, and only one showed the presence of foreign body within the myocardium of the left ventricle. Otherwise, no wall motion abnormality, or any sign of heart failure was detected.

After discussion among the cardiothoracic surgery team, exploration and removal of the foreign body was decided.

The patient was intervened with a median sternotomy approach, and the pericardium was opened and explored using cardiopulmonary bypass (CPBP).

**Figure 4:**
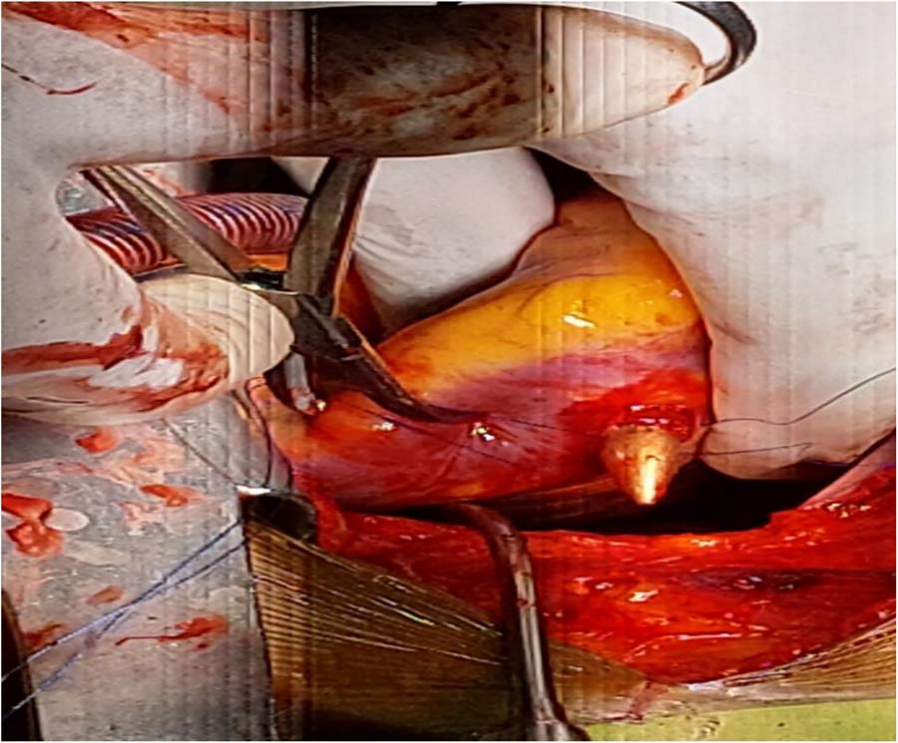
Partially extruded bullet located within the right ventricle wall.

**Figure 5:**
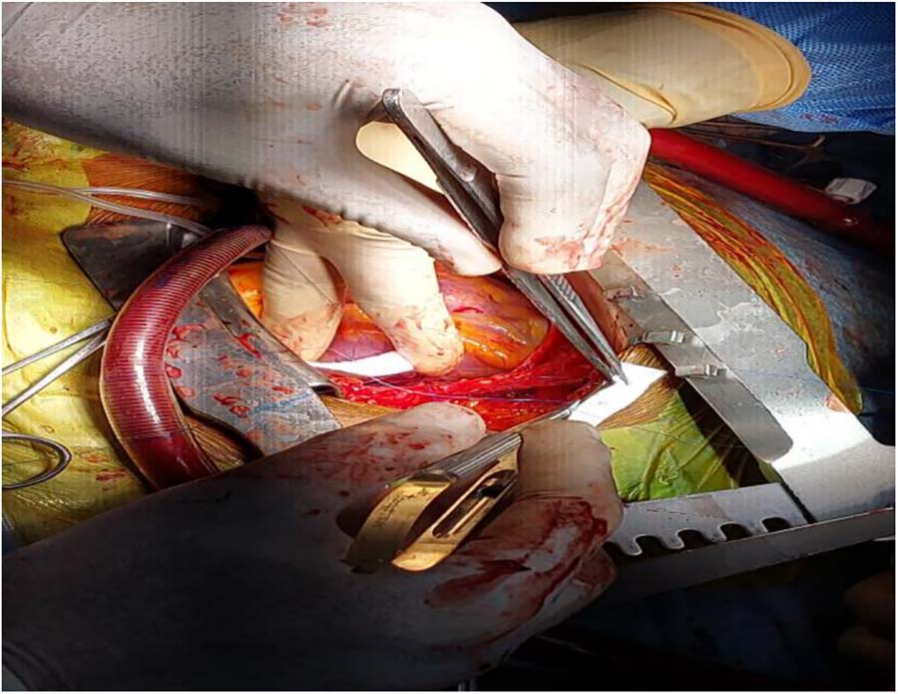
Picture depicting Teflon being used for closure of the defect.

There was a healed scar on the undersurface of the right ventricular wall deep in the myocardium close to the septum and posterior descending artery. The scar was opened, the bullet was extracted along with minimal purulent discharge, which was drained, the cavity was irrigated, and the defect was closed using artificial membrane polytetrafluoroethylene (PTFE) (teflon). Patient was weaned off the CPBP and regular heart beat was achieved. And the patient was extubated on table and was transferred to the cardiac intensive care unit (CICU) with stable condition and normal vital signs. The postoperative course of the patient was uneventful and he stayed a total of 7 days in the hospital after surgery, with 2 days in the CICU and the remaining in the regular ward. He was administered cefepime and vancomycin because of the intraoperatively detected purulent pericardial discharge. His postoperative echocardiography detected normal myocardial activity with no wall motion abnormality and no pericardial effusion. He was discharged on the seventh postoperative day with a stable condition. He was followed as an outpatient and has improved (Figs 4 and 5).

## DISCUSSION

Totally intramyocardial missiles are the rarest forms of cardiac projectile injuries, as the majority of such injuries are of partly myocardial with the remaining being either intracardiac or pericardial [3]. The most organized review of case reported and series of over 48 years showed that a half of cardiac projectile injuries are sharpnel injuries, and bullets contributed to only a fifth of the reported projectiles [3]. Furthermore, only 2 of the reported 45 collected case reports were purely intramyocardial bullets over the course of nearly half a century of a time period involving the second world war, the Vietnam war and the Korean war [3]. Our patient had a bullet as a mechanism of injury and totally myocardial location.

In general, projectile cardiac injuries and especially bullet injuries are highly fatal. The largest report showed a survival of only 23% in contrast with survival of 58% for stab injury patients [4]. Even worse survival rates are reported when considering patients that had cardiac arrest at the scene, *enroute* or at the emergency unit [4, 5].

In acute phase, projectile injuries generally present as either myocardial contusion, chamber laceration or perforation, valve or leaflet injuries, septal injuries or coronary artery injuries [6]. These patients generally present with exanguination, cardiac tamponade, tachyarrhythmia, heart block or cardiac arrest [3–8]. Few patients were reported to present with bullet embolization from the heart to peripheral organs [9]. These patients generally require surgical intervention to extract the bullet [4, 5]. None of these modes of presentation fits our patient’s clinical features.

Few patients present with non-specific symptoms or are asymptomatic at presentation [10, 11]. Similarly, in our patient no symptom or sign pertaining to the myocardial lodged bullet was detected. This is a scenario in which decision for surgery becomes challenging. Symbas and colleagues report showed the majority of the patients with no life threatening or significant symptoms with retained projectiles were treated non-operatively [3]. This and other reports [6] showed that conservative care is a valid component of the management of patients presenting with retained projectile [12]. To date no management guidelines could be made, but patients with no symptoms or only non-specific symptoms and no concerning investigative findings could potentially be treated non-operatively. But on peculiar finding in our patient is the presence of purulent fluid surrounding the bullet. This is the first time infectious complications are reported with a lodged myocardial bullet to our knowledge. We believe that in this particular case surgical intervention was in retrospect warranted.

## CONCLUSION

This is the first report of retained cardiac projectile missile reported from our country. The peculiarities of patients with retained bullet without symptoms and the controversies in the management remain. Individualized approach to such cases is warranted.

## CONFLICT OF INTEREST STATEMENT

None declared.

## FUNDING

No funding was acquired for this case report.

## CONSENT FOR PUBLICATION

Written consent for publication was acquired from the patient.
